# Limits of Active Laser Triangulation as an Instrument for High Precision Plant Imaging

**DOI:** 10.3390/s140202489

**Published:** 2014-02-05

**Authors:** Stefan Paulus, Thomas Eichert, Heiner E. Goldbach, Heiner Kuhlmann

**Affiliations:** 1 Institute of Geodesy and Geoinformation, Chair of Geodesy, University of Bonn, Nußallee 17, Bonn 53115, Germany; E-Mail: heiner.kuhlmann@uni-bonn.de; 2 Institute of Crop Science and Resource Conservation, Division Plant Nutrition, University of Bonn, Karlrobert-Kreiten-Str. 13, Bonn 53115, Germany; E-Mails: t.eichert@uni-bonn.de (T.E.); h.goldbach@uni-bonn.de (H.G.)

**Keywords:** high resolution 3D laser scanning, plant leaves, chlorophyll, optical properties of leaf surfaces

## Abstract

Laser scanning is a non-invasive method for collecting and parameterizing 3D data of well reflecting objects. These systems have been used for 3D imaging of plant growth and structure analysis. A prerequisite is that the recorded signals originate from the true plant surface. In this paper we studied the effects of species, leaf chlorophyll content and sensor settings on the suitability and accuracy of a commercial 660 nm active laser triangulation scanning device. We found that surface images of *Ficus benjamina* leaves were inaccurate at low chlorophyll concentrations and a long sensor exposure time. Imaging of the rough waxy leaf surface of leek (*Allium porrum)* was possible using very low exposure times, whereas at higher exposure times penetration and multiple refraction prevented the correct imaging of the surface. A comparison of scans with varying exposure time enabled the target-oriented analysis to identify chlorotic, necrotic and healthy leaf areas or mildew infestations. We found plant properties and sensor settings to have a strong influence on the accuracy of measurements. These interactions have to be further elucidated before laser imaging of plants is possible with the high accuracy required for e.g., the observation of plant growth or reactions to water stress.

## 1. Introduction

Three-dimensional measurement and analysis of plant properties offer great benefits for the understanding of plant responses to biotic and abiotic stresses [1]. On the level of single organs this can be done by using stereo camera systems [2] or laser scanning [3,4]. The laser technique is particularly suitable for tracking the smallest tissue deformations and monitoring plant development due to its quick and high resolution data collection [4]. Producing 3D point clouds in the Euclidian space laser scanning allows the calculation of structural and geometric parameters like canopy height and structure to monitor plant growth and shape responses [1]. These measurements are limited to the working range of the scanning device. Laser scanning requires a trade-off between range, which means the distance between scanner and object, and resolution, the distance between two measured 3D values.

Ground-based scanning systems measure in distances between 1–100 m and were used, e.g., for characterizing the stem geometry of trees [5] or 3D modeling of a tomato canopy [4]. They provide a minimal resolution of around 0.5 mm [1]. Laboratory scale triangulation sensors resolve much smaller details as shown by [6]. These industrial measuring combinations consist of an articulated measuring arm and a mounted laser scanning device and enable resolutions of about 10 μm. This allows the extraction of plant-organ specific parameters as well as the identification of organs in the recorded point cloud. Using such instrumentation, tracking of individual plant development becomes realizable more detailed and more accurately than it can be done with current stereo camera based techniques as e.g., used by [3] or [4]. Conventional camera systems raise the problem of fusion, which means the combination of different points of view. This requires the use of scanning targets to have reference points in every view and thus increases the workload and reduces the amount of recordable data. In contrast, measuring arms or laser trackers scan single points with high geometric accuracy leading to more precise results.

**Figure 1.**
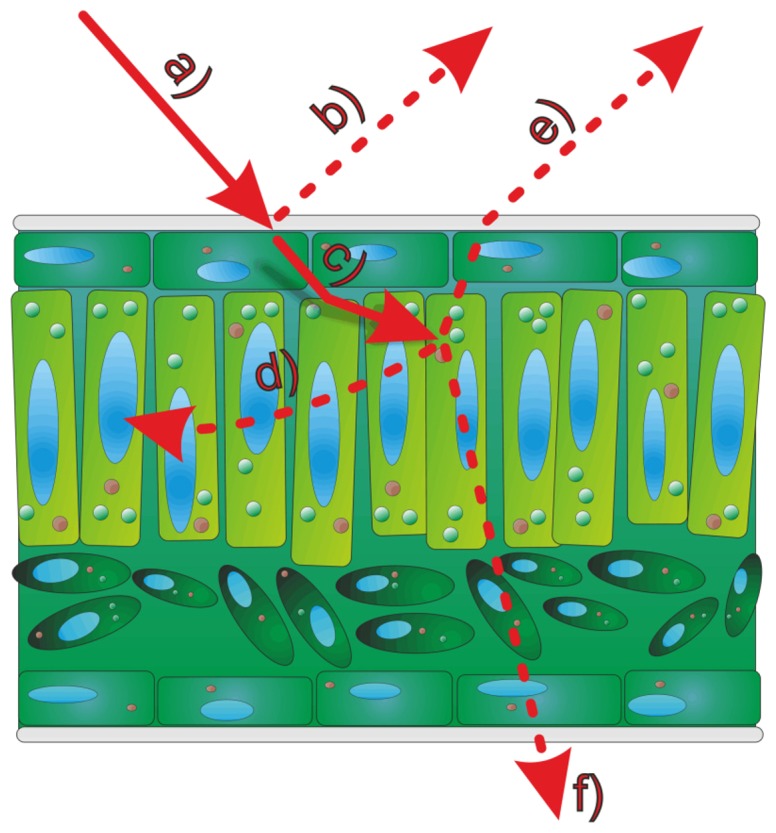
Interactions between a light beam and a leaf surface. The light beam (**a**) is partly reflected on the epidermal layer (**b**) and partly entering the tissue (**c**). Underneath the surface its energy can be absorbed by chlorophyll in the mesophyll (**d**) scattered back to the surface (**e**) or transmitted through the spongy tissue (**f**).

The most accurate measurements shown in literature [4,6,7] used wavelengths of about 660–680 nm which fall within the absorption maximum range of chlorophyll. It has been shown by [8] that the chlorophyll concentration of leaves has a noticeable effect on the measurements of terrestrial laser scanning systems using a green (532 nm) laser. Any interaction between leaf chlorophyll and laser scanning indicates that the leaf interior affects the measurement, *i.e.*, that the recorded 3D information is affected at least partly by structures underneath the leaf surface due to penetration of the laser light. Furthermore, epidermal cells lack chlorophyll, and considering that in many plant species the epidermal layer may account for a substantial proportion of the leaf thickness it is therefore of particular interest to investigate if the impinging laser beam (Figure 1a) is reflected by surface structures (Figure 1b) or penetrates this cell layer (Figure 1c). The penetrated beam can be absorbed (Figure 1(d)), scattered back to the leaf surface (Figure 1e) or transmitted through the leaf (Figure 1f).

This leads to the question whether it is at all possible to image the true surface of a plant or a plant organ with lasers emitting light in the absorption range of chlorophyll. In this context, true surface denotes the surface which separates the plant material from its surrounding medium. This is, however, a crucial prerequisite for the realistic imaging of geometric plant structures or the detection of smallest deformations in plant organs due to growth or interaction with stress. The objective of this study was thus to test a current commercialized laser scan system for its suitability to image plants with high (sub-millimeter) accuracy.

**Figure 2.**
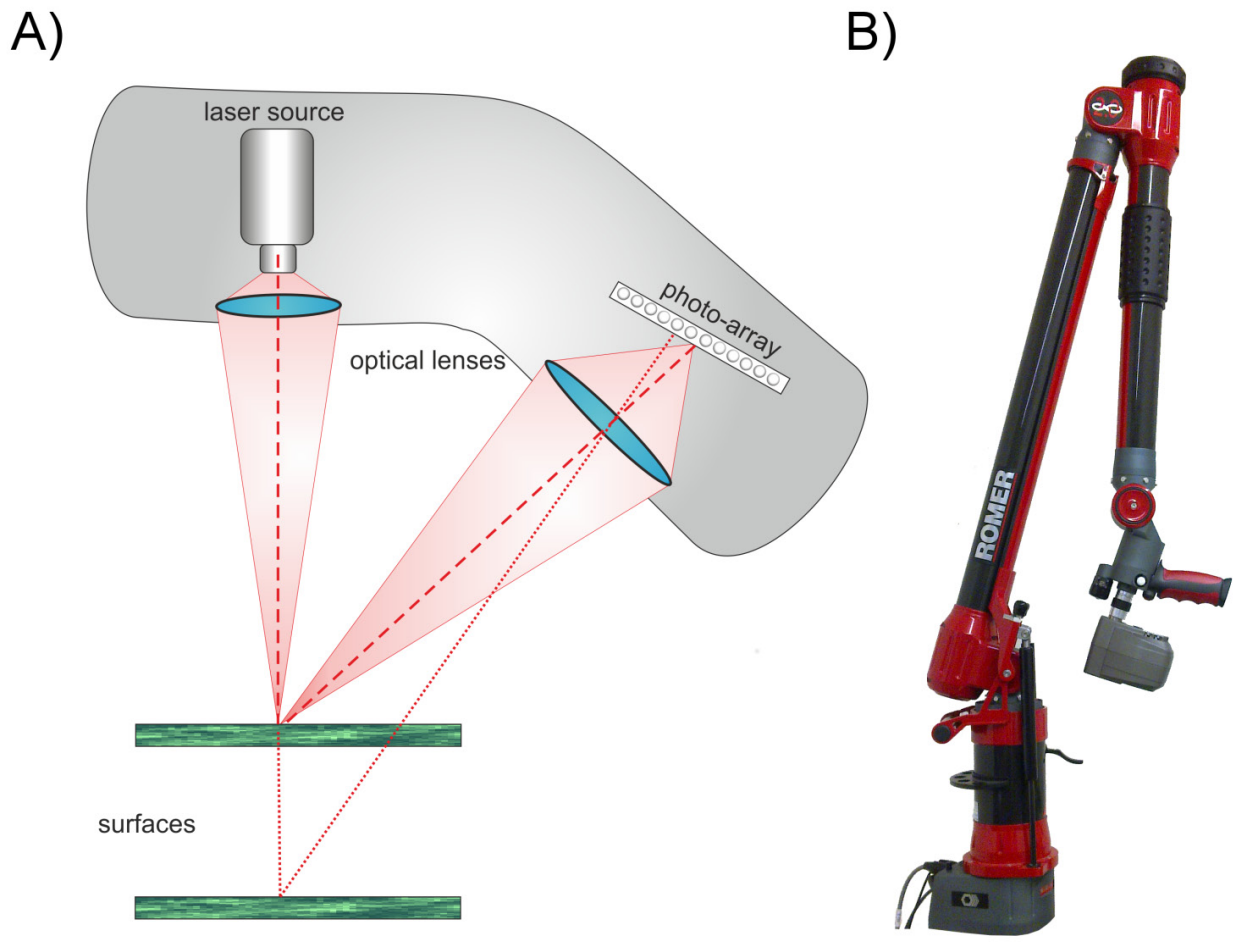
Simplified illustration of the active laser triangulation technique (**A**) used in the measuring arm—laser scanner combination (**B**). This combination enables measurements with a resolution of 14 μm and an accuracy of 45 μm.

We used an active triangulation laser scanner (660 nm) combined with an articulated measuring arm (Figure 2) to study the interactive effects of sensor settings (exposure time) and leaf properties (surface roughness, chlorophyll concentration, diseases like mildew) on the imaging of plant leaves. The conditions of our experiments were chosen in a way that any occurring inaccuracy can unambiguously be attributed to the laser scanner itself. For this purpose we conducted the experiments under dim light and used detached leaves or isolated epidermal strips where necessary.

## 2. Methods

### 2.1. Measuring Setup

Our experiments were performed using a combination of an articulated measuring arm (Romer Infinite 2.0, Hexagon Metrology Inc, North Kingstown, RI, USA) and a laser line scanner device (Perceptron V5, Perceptron Inc. Plymouth, MI, USA, Figure 2). This combination provides a Euclidian 3D point cloud with a resolution of 14 μm (point to point distance) supporting an accuracy of up to 45 μm for the combined laser lines [9,10].

The laser scanner used active laser triangulation for 3D line imaging. The principle is shown in Figure 2A. An emitted laser line (660 nm) is reflected at the surface of the object. This reflection is recorded by a photosensitive array (e.g., a CCD array). The location of the received signal on the photosensitive array depends on the distance between scanner (reference plane) and the reflecting surface. By using a defined geometrical arrangement of laser emitter and CCD sensor, the position of the laser line can be interpreted as a distance measure [11]. Due to this, the scannable volume is limited to the area visible to the CCD array. In our case this scanning field has a depth of 110 mm and a width of 93–140 mm. For a single line scan, as it is not influenced by the articulated measuring arm, we can assume that the accuracy definition of the manufacturer of 24 μm is valid [10]. This can be defined by two manufacturer implemented thresholds. A scan is rejected, if the scanline is above a certain width or the recorded laser intensity is under a certain limit. Low energy is caused by an absorption of the energy or objects with unsuitable reflection properties. High energy is caused by specular reflections on the object's surface. For scanning the width of the laser line is reduced to its centre of gravity (CoG) and the 3D coordinates for the complete line are extracted simultaneously. The scan speed is at is maximum 60 Hz and has to be adjusted, if choosing the maximum exposure time, down to 30–40 Hz. This results in a scan time of five to ten seconds for e.g., a cherry laurel leaf. The resulting point cloud consists of pure XYZ data without any additional information like reflectance.

Due to a fixed imaging geometry between laser source and CCD sensor, the laser scanning unit measures only changes in distance between sensor und surface. Horizontal movements of the laserline are not imaged. Thus, motion blur, as it is known from 2D cameras is not possible.

The combination of the laser scanner with a measuring arm (Figure 2B) enables a spherical measuring volume of 1.4 m radius [9]. Because laser scanning only measures the distance of the object's surface referred to the scanner reference plane, the single scan line has to be composed to the final point cloud. Hereafter image denotes the graphical presentation of the measuring values in 3D and 2D.

Coupling a laser scanner and a measuring arm enables an automated fusion of the scanned laser lines. This is done using high precision angle encoders to calculate the position and orientation of the scanner for every scan line. To account for the different reflection behavior of surfaces the camera exposure time can be adapted. This parameter is factory-implemented as a parameter without unit ranging from 0–2,100 and includes the exposure time, the automatic readout and the transfer to the processing unit. The exposure times used in our experiment correspond to the factory adjusted measuring profiles constructed to scan industrial material like wood or metal with different reflecting surfaces [10]. For quality reasons standard scanning properties concerning laser line and CCD intensity settings were used as provided and suggested by the company. For comparability reasons, the scans were performed consistently in vertical direction over the object with a uniform, slow speed and mainly in one direction. The single scans of an experiment correspond to the same coordinate system and can be fused lying on top of each other. All the experiments were conducted in the basement laboratory with dimmed light condition.

### 2.2. Plants

Leek (*Allium porrum* L.) was bought at a local market. Variegated weeping fig (*Ficus benjamina* L. cv. Starlight) and cucumber (*Cucumis sativus* L. cv.) were grown in a greenhouse at the Department of Plant Nutrition, University of Bonn. Cherry laurel (*Prunus laurocerasus* L.) leaves of different physiological states and senescent Honeysuckle (*Lonicera spec.* L) leaves were collected in autumn 2011 in the front garden of the Institute of Geodesy and Geoinformation, University of Bonn. Sword fern (*Nephrolepis exaltata* (L.) Schott) leaves growing as an ornamental plant in the Plant Nutrition Department (University of Bonn) were used for chlorophyll extraction.

#### Experiment 1

Variegated *F. benjamina* leaves were removed from the plant, trimmed to fit on a microscope slide and fixed using adhesive tape. The leaves were scanned repeatedly with increasing exposure times ranging from 0 to 2,100.

#### Experiment 2

To study the effect of chlorophyll concentration on the scanner signal, leaf pigments were extracted from *N. exaltata* leaves with 80% acetone using a mortar and a pestle. To assist extraction and to buffer the extract, small amounts of sea sand and CaCO_3_ were added. The extract was filtered (Whatman No.1) and used immediately. Pieces of filter paper (approximately 2 cm × 2 cm, Whatman No.1) were soaked with the extract and after evaporation of the acetone this procedure was repeated one to nine times to obtain a series of increasing chlorophyll (a + b) concentrations ranging from 0 (no extract applied) to approx. 100 μg·cm^−2^. After scanning the dry filter papers repeatedly with different exposure times (0, 20, 80, 420 and 2,100), the pigments were desorbed from the filter paper using 10 mL of 80% acetone and chlorophyll a and b as well as carotinoids were determined by the method of [12].

The density of pixels (area covered by pixel in percent of total area) was determined by using image processing. A circle was fitted inside the scanned chlorophyll area. The amount of scanned pixels was considered with regard to the complete area of the fitted circle to obtain a measure for the density of the covered pixels in the circle. This calculation was performed using Matlab 2009b (MathWorks Inc., Natick, MA, USA). The relationship between chlorophyll concentrations and pixel density was calculated using linear and non-linear regression (SigmaPlot, for Windows, Version 10.0, Systat Software Inc., Chicago, IL, USA).

#### Experiment 3

Leaves of *P. laurocerasus* were picked in autumn, when leaves at different stages of senescence were present on the plants. Leaf strips were cut, mounted on glass slides and scanned repeatedly with increasing exposure times. Senescent leaves of *Lonicera spec.* with green, chlorotic and necrotic areas were picked and scanned at different exposure times with no further preparation.

#### Experiment 4

Epidermal strips (ES) were peeled from *A. porrum* leaves, mounted on glass slides previously partially sprayed with highly reflective scan-spray (3D Scanspray, T.HS Gbr, www.scanspray.de) and fixed with adhesive tape. The ES were often contaminated by small patches of mesophyll still adhering to the epidermal cells. This was utilised as an indicator of signals originating from below the ES.

The ES were scanned repeatedly with increasing exposure time. Data were analysed by Geomagic Studio 12 64 bit (Geomagic, Morrisville, NC, USA). The scans were positioned and the relevant areas were separated from the background of the surrounding scan, followed by a vertical exaggeration and the visualization for the side cuts.

#### Experiment 5

This experiment was designed like Experiment 4 with the following variations: to start of a grid of lines was painted on the glass slide with a permanent marker to be able to recognize signals originating from beneath the ES, then up to five ES were stacked and scanned with exposure times of 80 or 2,100.

#### Experiment 6

*Cucumis sativa* ‘Vorgebirgstraube’ plants were manually infected with mildew (*Spaerotheca fugliginea*) at growth stage (GS) 10. When the third leaf was unfolded at the main stem (GS 13) the plants had developed visible infested areas. The leaves were scanned using exposure times ranging from 5 to 2,100.

## 3. Results and Discussion

In many species leaves represent the major proportion of aboveground biomass. We therefore used detached leaves to study the feasibility of laser triangulation for the measuring of plant surfaces. We selected leaves from different species depending on the specific research questions and on their availability. The images shown in the following sections are the visualisation of the 3D point clouds recorded by the sensor. Every data point recorded in Euclidian space is represented by a black point. Neither point cloud meshing algorithms nor surface smoothing have been performed; instead the original 3D point cloud was directly evaluated. The laser scanner uses a triangulation technique which means that a laser line is emitted and the reflection from the surface of the object is measured on a photosensitive array.

Dependent on the distance between sensor and object the backscattered laser line is displaced on the sensor array. The composition is done automatically by the sensor. Each line scan represents a single viewpoint. The camera exposure time or shutter time is implemented as a parameter without unit ranging from 0–2,100.

The sensor has two implemented thresholds. A measurement point is rejected, if the scan line is, at this point, above a certain width or the recorded laser energy is under a certain limit. To derive the 3D information of one point of the laser line, the center of gravity (CoG) is calculated column by column, using the illuminated pixels of one column. A violation of the maximum width means that too many pixels have an influence on this CoG calculation. In this case the accuracy of the measurement cannot be guaranteed and the pixel measurement is rejected. An overexposure leads to a wide scan line, which is similar to a blurring effect. A rejected signal manifests in a missing black pixel in the image. Further reasons for an increased width of the laser line can be penetration effects that cause widening of the laser line as it occurs when scanning e.g., marble.

### 3.1. Experiment 1: Origin of the Signal Relative to the Leaf Surface

In this experiment we tested the hypothesis that, by selecting appropriate exposure times, it is possible to image the true surface of leaves. To evaluate this hypothesis we used variegated *F. benjamina* leaves. The basic idea was that in the case of true surface imaging the chlorophyll concentration of the underlying mesophyll should not affect the detected signals.

**Figure 3.**
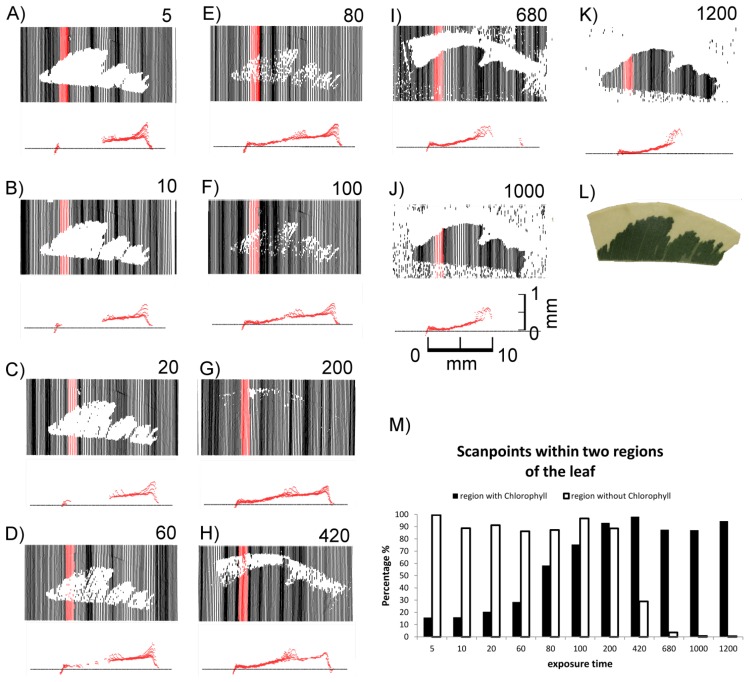
Effect of exposure time on images of a variegated *F. benjamina* leaf. Top views show recorded pixels in black or red. The corresponding cross sections below show pixels recorded in the red parts of the top views. Exposure times were: 5 (**A**), 10 (**B**), 20 (**C**), 60 (**D**), 80 (**E**), 100 (**F**), 200 (**G**), 420 (**H**), 680 (**I**), 1,000 (**J**), 1,200 (**K**). A RGB image is shown in (**L**). For differentiation reasons the vertical distances in the cross sections are exaggerated by factor 5. A quantitative evaluation is shown in subfigure (**M**).

As shown in Figure 3, the laser scanner recorded different images depending on the sensor exposure time. Differences in the distances between the scan lines of the top views can be explained by the hand-operated design of the scanner and the human-influenced scanning speed. At lower exposure times (0–10) exclusively the non-green areas were imaged in the top views (Figures 3(A–D)), whereas at high exposure times (680 and higher) only green areas were recorded (Figures 3(K–O)). In the intermediate exposure time range there was a transition of these two types of images (Figures 3(E–J)). Figure 3 also shows cross sections of the red areas marked in the top views. Figure 3(M) describes the covering of the two areas (green and white) with scanned points in a quantitative evaluation. Scans below exposure time 5 and over 1,200 have been omitted due to no visible change of effects. At an exposure time of 200 both regions can be imaged almost completely with more than 90% (3 G&M).

**Figure 4.**
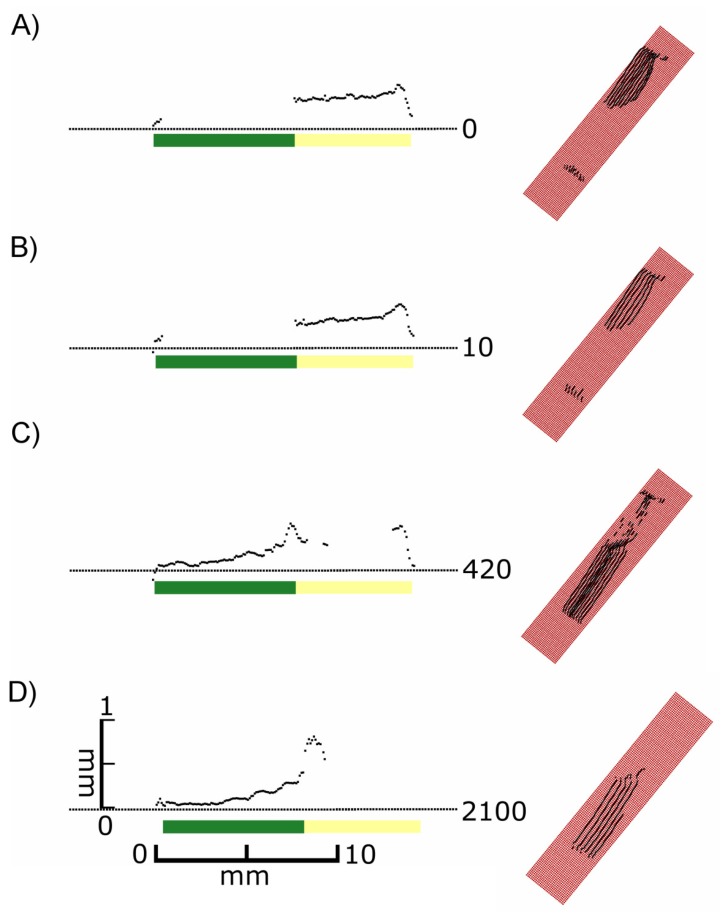
Side views of selected individual scan lines (**Left**) and 3D views of the surrounding array of scan lines (**Right**) obtained from a variegated *F. benjamina* leaf scanned at different exposure times. The side views were chosen from the middle region of the array of scan lines shown on the right. The coloured bars indicate the distribution of the green and yellow leaf area. The 3D views (right) show the surface of the underlying glass slide in red and the scanned lines in black. The vertical distances in the cross sections are exaggerated by factor 5.

It became evident that in many cases the recorded signals originated from various depths in relation to the leaf surface, *i.e.*, the true surface structures were not always imaged. This is shown in detail in Figure 4. At exposure times of 5 or higher “ghost pixels” were recorded in the transition zone between green and chlorophyll-free leaf areas apparently originating from above the true leaf surface (Figures 3C–O and 4B–D).

These findings show that in *F. benjamina* leaves the image reflects sub-surface characteristics rather than the surface itself and that the quality of the recorded image strongly depends on the exposure time parameter selected for scanning. The presence of chlorophyll had a intense effect on the laser scanning results after the laser beam penetrated the epidermis layer. Therefore we conducted further experiments aiming at the effects of surface and subsurface structures on the scanned images of leaves.

### 3.2. Experiment 2: Interactive Effects of Chlorophyll Concentration and Sensor Exposure Time on the Quality of the Image

The following experiment was conducted to study the interaction between exposure time and chlorophyll concentration on a more quantitative basis. It is known that the potential of leaves to absorb light radiation, especially in the wavelength range between 400 and 700 nm, depends on the content of chlorophyll pigments. For our experiment pieces of filter paper were impregnated with chlorophyll extracted from fern leaves to obtain a series of increasing chlorophyll concentrations. These “artificial leaves” were scanned with different exposure times.

**Figure 5.**
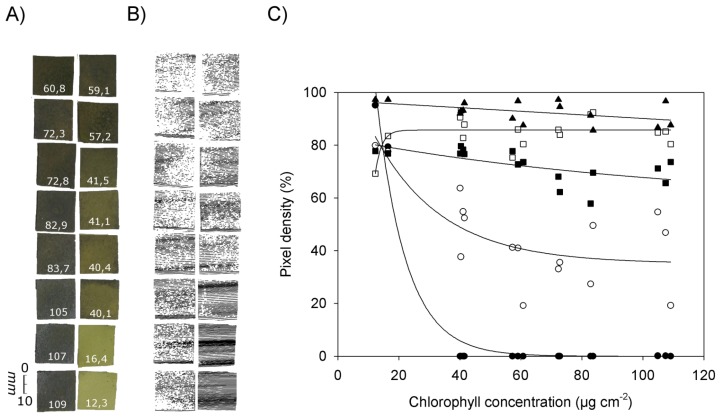
Relationship between the chlorophyll concentration and the pixel density in laser scans at different exposure times. Pieces of filter paper were impregnated with chlorophyll extracts obtained from fern leaves by acetone extraction and pixel density of the images scanned with different exposure times was measured. (**A**): RGB images of the pieces of filter paper (**B**): corresponding image obtained by laser scanning with exposure time 20. (**C**): Relationship between pixel density and chlorophyll concentration. Filled circles ●): exposure time = 0, open circles ○): exposure time = 20, ■ filled squares: exposure time = 80, open squares □: exposure time = 420, filled triangles ▲: exposure time = 2,100. The white numbers depict the chlorophyll concentration of every tested patch in μg·cm^−^².

Figure 5 shows RGB images of pieces of filter paper with chlorophyll concentrations ranging from 12.3 μg·cm^−2^ to 109 μg·cm^−2^ (Figure 5A), the corresponding images obtained by laser scanning at an exposure time of 20 (Figure 5B), and the relationship between chlorophyll concentration and pixel density in the laser scans at different exposure times (Figure 5C). Missing pixels within a scan or an individual scanned line (Figure 5B) can be explained by chlorophyll absorption of the laser light and/or by scattering of the laser beam resulting in less energy reaching the CCD camera.

Figure 5C shows that at chlorophyll concentrations below 20 μg·cm^−2^ all exposure times resulted in relatively high pixel densities between ca. 70% (exposure time 420) and 97% (exposure time 2,100). At an exposure time of 80 or lower pixel density decreased with increasing chlorophyll concentrations. This tendency was the more pronounced the lower the exposure time was chosen. At the smallest exposure time (0) the pixel density already reached 0% at chlorophyll concentrations of 40 μg·cm^−2^. The best fit of the data was obtained with an exponential decrease (y = 361.5 × e^−0.103x^, R^2^ = 0.975, p < 0.0001). At an exposure time of 20 the decrease in pixel density was less pronounced than at an exposure time of 0, and values showed a high variability (y = 35.1 + 83.6 • e^−0.0448x^, R^2^ = 0.606, p < 0.003). At an exposure time of 80, pixel density decreased slowly when the chlorophyll concentration was increased (y = 57.5 + 25.3 × e^−0.0090x^, R^2^ = 0.409, p < 0.033). With an exposure time of 420 best fit was obtained with the equation y = 85.9 (1 – e^−0.154x^) (R^2^ = 0.367, p < 0.013), and with an exposure time of 2,100 a linear equation produced best fit (y = 97.0 − 0.068x, R^2^ = 0.246, p < 0.051).

These results corroborate the previous finding that the images obtained by laser scanning are highly affected by the presence of chlorophyll. With a sufficient long sensor exposure time a complete image can be recorded as long as the exposure time is adapted to the chlorophyll content. The higher the chlorophyll concentration, the higher the exposure time has to be selected to obtain a clear scanning result.

### 3.3. Experiment 3: Characterising the Physiological Status of Plants by Combining Scans with Specific Exposure Times

The previous experiments indicated that the scanning results of leaves depend on the selected exposure time. As demonstrated in Figures 3 and 4 either green or white parts of leaves were recorded, depending on exposure time. Figure 5 also shows that for each exposure time there was a different quantitative functional relationship between the scanning signals and the chlorophyll concentration of the tissue. This prompted us to test the hypothesis that images created with different exposure times can be utilised to characterize the physiological status of leaves with different chlorophyll concentrations. To test this hypothesis we obtained scans of senescent cherry laurel leaves and honeysuckle leaves with gradual progress of leaf senescence at different exposure times.

**Figure 6.**
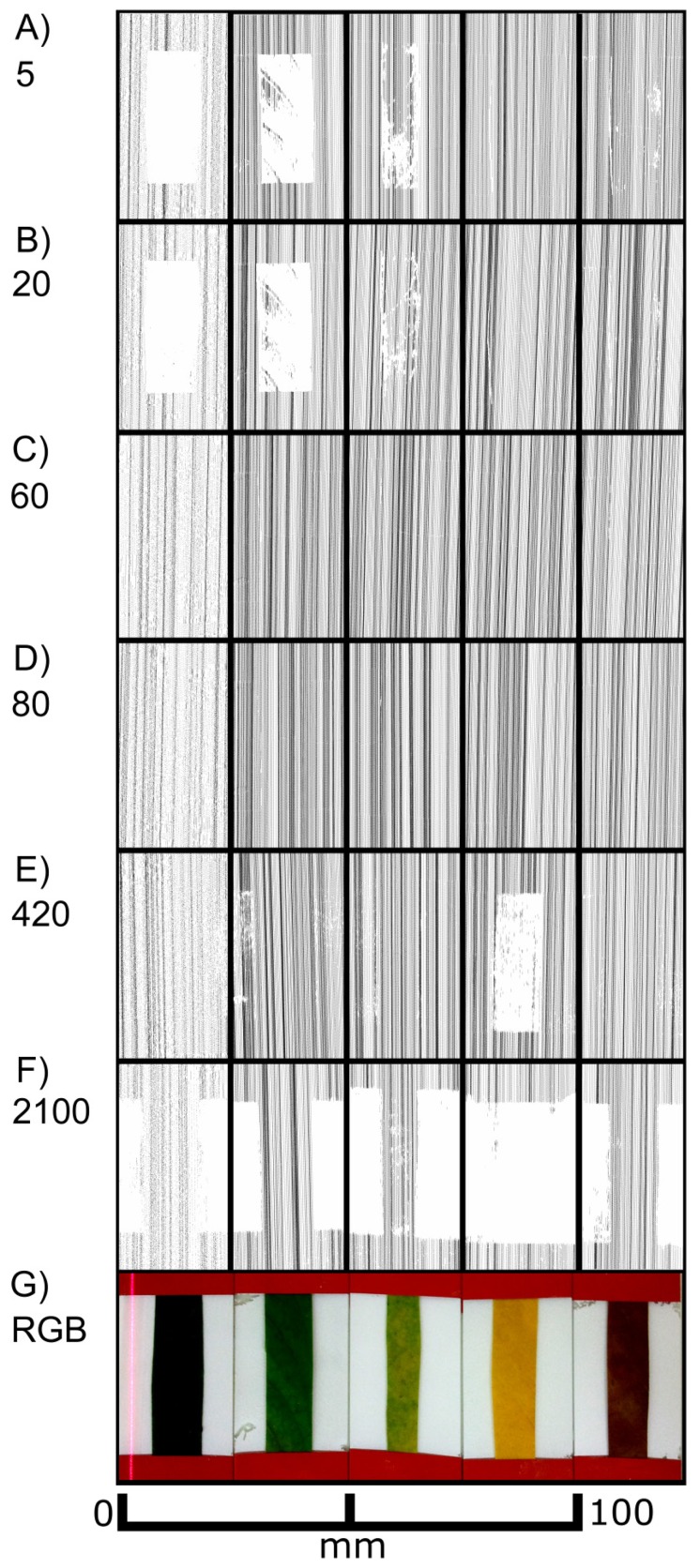
Top views of *P. laurocerasus* leaves in different stages of senescence (from left to right: dark green, medium green, light green, yellow and brown). **(A**–**F)**: Laser scans with exposure times as indicated on the right side. (**G**): RGB image.

Figure 6G shows RGB images of cherry laurel leaves in different phases of leaf senescence, *i.e.*, with green, chlorotic and necrotic areas, together with the corresponding images obtained by laser scanning with different exposure times. At low exposure times (20 or lower) areas with high chlorophyll concentrations could not be imaged, whereas with the highest exposure time areas with low chlorophyll concentrations could not be imaged (yellow leaf in Figure 6). Brownish, necrotic leaf tissue could be detected irrespective of exposure time. Together, these results indicate that scanning of cherry laurel leaves at different exposure times allows the classification and identification of different states of senescence. Areas which give no signal in the form of recorded pixels at low exposure time but do so at high exposure times are high in chlorophyll, whereas areas showing the opposite effects, *i.e.*, a signal at low exposure times and no signal at high exposure times are chlorotic. Areas resulting in signals irrespective of the exposure time can be classified as necrotic.

**Figure 7.**
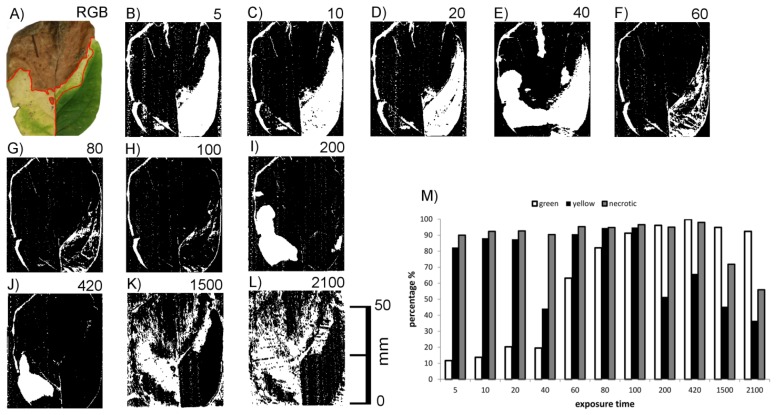
RGB image of a senescent *Lonicera spec.* leaf and corresponding laser scanned images. RGB image of a senescent *Lonicera spec.* leaf (**A**) corresponding laser scanned images at different exposure times. (**B**–**L**). Exposure times were 5 (**B**), 10 (**C**), 20 (**D**), 40 (**E**), 60 (**F**), 80 (**G**), 100 (**H**), 200 (**I**), 420 (**J**), 1,500 (**K**) or 2,100 (**L**). The leaf was fixed on a background of white paper. A quantitative evaluation is shown in subfigure M.

This preliminary result obtained with honeysuckle leaf sections was checked using honeysuckle leaves with green, chlorotic and necrotic regions. An example is shown in Figure 7. It shows the connection between exposure time and the ability to image the leaf surface Figure 7B–M with a quantitative description for the three regions. Scans below exposure time 5 have been omitted due to no visible change of effects. Again, the green leaf area did not return signals at low exposure times (<80) and the chlorotic areas was in many cases invisible at high exposure times (>100). The necrotic upper part of the leaf was more or less visible at all exposure times.

The effects of exposure time were, however, not as homogeneous as with cherry laurel leaves. At the highest exposure times, for example, the leaf veins interfered with the effects of chlorophyll causing signals within the otherwise invisible chlorotic area, and some signals were missing in the necrotic area. Cherry laurel leaves (Figure 6) have a smooth surface whereas honeysuckle leaves are heavily structured due to leaf veins and shrivelling in the necrotic parts. We thus hypothesized that surface structuring may also have strong effects on the imaging of leaves.

### 3.4. Experiment 4: Effects of Surface Roughness on Images Obtained by Laser Scanning

The previous results showed that images recorded by laser scanning may be strongly influenced by chlorophyll in the subsurface tissue (Figures 3, 4 and 5). We have shown that this effect can be applied to characterise the physiological status of a leaf (Figures 6 and 7). In the last experiments with honeysuckle we found evidence of interfering effects on surface roughness. Therefore we conducted additional experiments with leek leaves which are characterised by a rough, structured surface and additionally densely covered by epicuticular waxes.

**Figure 8.**
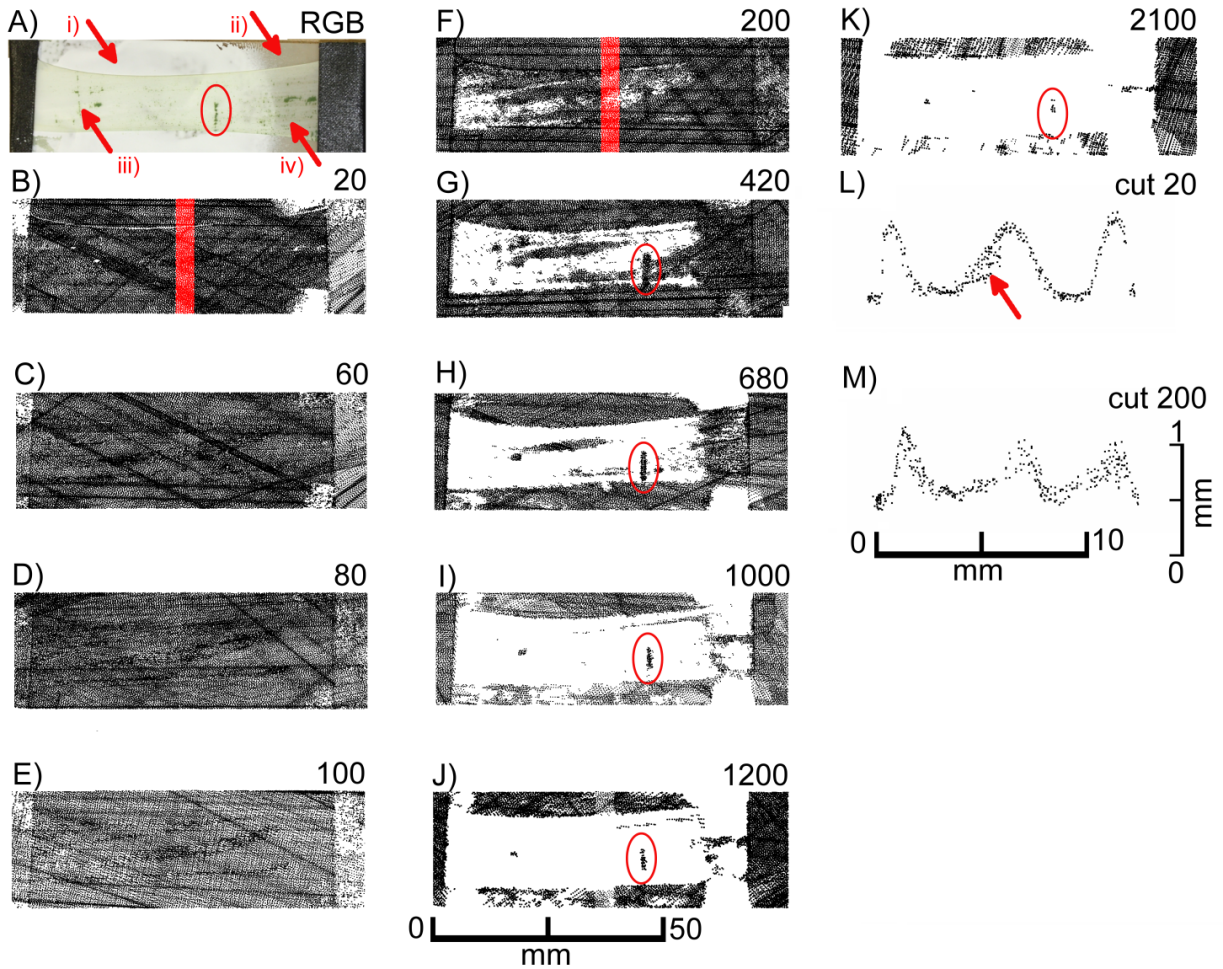
Top views of laser scans of *A. porrum* epidermal strips mounted on a glass slide (**A**) at different exposure times (**B**–**K**), side views of images obtained at an exposure time of 20 (**L**) and 200 (**M**). Exposure times were 20 (B), 60 (C), 80 (D), 100 (E), 200 (F), 420 (G), 680 (H), 1,000 (I), 1,200 (J) or 2,100 (K). The red marked areas in (B,F) correspond to the pixels shown in L and M, respectively. The vertical distances in the cross sections are exaggerated by factor 5 (L,M). The regions with the red arrows in subfigure (A) denote the different regions of underlying scanspray (i), clear glass carrier (ii) and epidermis under test with underlying scanspray (iii) and without underlying scanspray (iv).

We used isolated leek epidermal strips mounted on glass slides and performed scans at different exposure times. Figure 8 shows the top views of the laser scans as well as selected cross sections from scanning exposure times of 20 and 200 extracted from the areas marked red in the top views. At low exposure times (up to 200) images (black pixels) representing the leaf surface were obtained, while the signal faded out progressively at exposure times of 420 and above. As in *F. benjamina* leaves, chlorophyll containing mesophyll cells, which were still adhering to the epidermis in some small patches (red ellipse), produced a signal at high exposure times indicating that the leaf surface was penetrated and the laser light was reflected by the mesophyll. At high exposure times the reflective layer of “scan spray”, which covered the major part of the underlying glass slide, failed to produce signals in the regions underneath the epidermis (region iii), while in areas not covered by the epidermis (region i) pixels were detected. This indicates that the laser light was probably scattered (and maybe partly absorbed) by the epidermal cells. An overexposure can be denied due to the scanned pixels in region iii, scan spray can be denoted to be best reflecting.

A comparison of cross sections obtained at low exposure time (20, Figure 8L) and intermediate exposure time (200, Figure 8M) reveals that at low exposure times a much sharper image was obtained than at intermediate exposure times. With one exception (marked with an arrow in Figure 8L) a rather thin line of pixels was obtained with the low exposure time, whereas at the high exposure time a cloud of pixels was obtained representing signals not only from the leaf surface but mostly from underneath the leaf surface. This can be interfered not only from the blurring of the pixels but also from the less pronounced topography of the epidermal structures. The epidermal fold which can be seen in the left part of the images is, for example, much higher when scanned at low exposure times (Figure 8L) than at high exposure times (Figure 8M).

### 3.5. Experiment 5: Effects of Chlorophyll Free Leaf Tissue on Laser Scanning of Leaves

The previous experiment indicated that at low exposure times the structured surface of leek leaves could be imaged by laser scanning, while at high exposure times no signals were recorded except in areas were some chlorophyll containing mesophyll was present. This indicates that at high exposure times the chlorophyll-free epidermal tissue is virtually transparent and cannot be imaged at all. Considering that in some species, e.g., in *Nerium oleander*, leaves are covered by multiple epidermal layers, this may have a strong effect on the accuracy of measurement of leaf properties such as leaf thickness and derived parameters such as plant volume or biomass. We therefore studied the reflectivity of multiple layers of leek epidermal strips at different exposure times. To be able to identify signals originating from the underlying glass slide we used a grid of black stripes drawn with a permanent marker.

**Figure 9.**
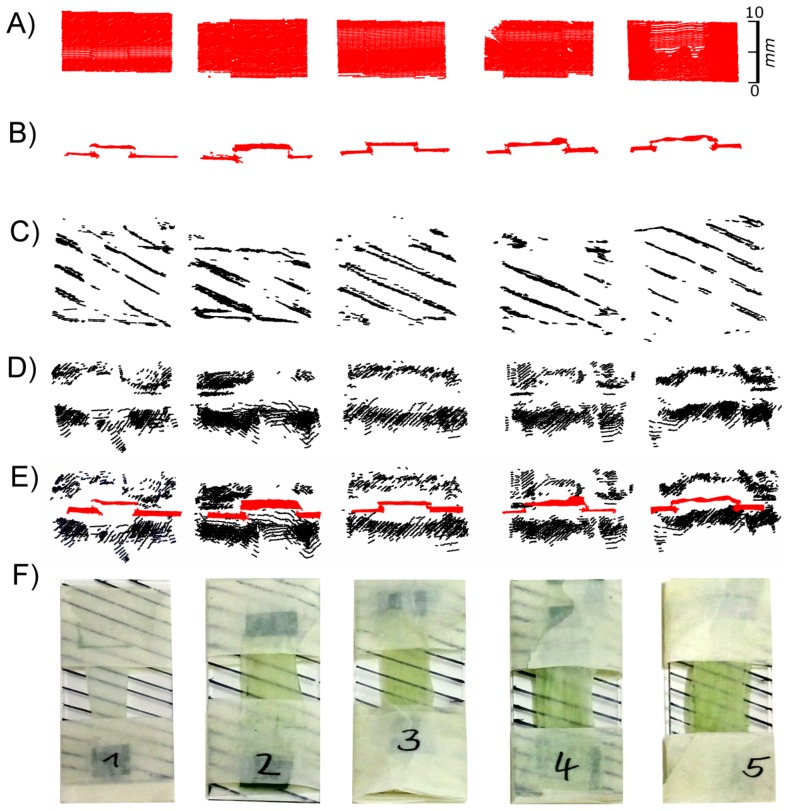
Images obtained by laser scanning of epidermal strips obtained from A. porrum at different exposure times. Images obtained by laser scanning of epidermal strips obtained from *A. porrum* with an exposure time of 80 (**A** from above, **B** from the side, and the red data in **E**) or 2,100 (**C** from above and **D** from the side). From left to right the number of stacked epidermal strips increased from 1 to 5 as shown in the RGB images.

Figure 9 shows images obtained at low and high exposure times. While at low exposure times images of the epidermal surfaces were recorded (Figures 9(A, B)) this was not the case with a high exposure time (Figure 9C,D). With high exposure times clearly false images were recorded. The pattern of black stripes also appeared above the surface of the epidermal strips (Figures 9D,E), whereas no signal was obtained from the leaf surface.

### 3.6. Experiment 6: Effects of Surface Modifying Diseases on Laser Scanning of Leaves

**Figure 10.**
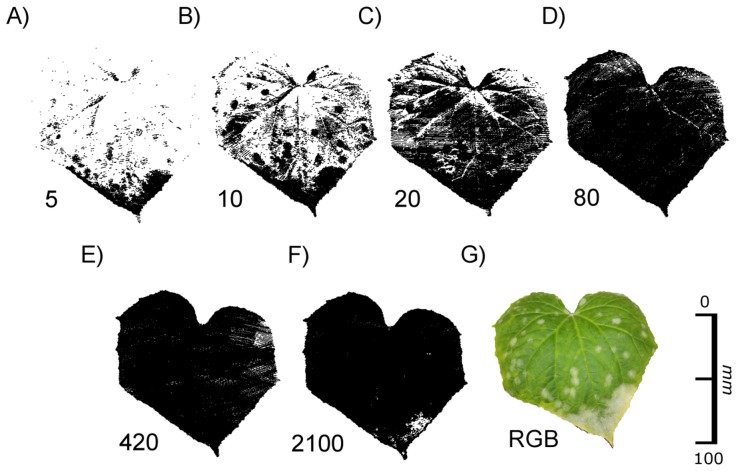
Laser scanning images of a mildew infected cucumber leaf recorded with different exposure times. Exposure times were 5 (**A**), 10 (**B**), 20 (**C**), 80 (**D**), 420 (**R**), 2,100 (**F**). Image G shows the corresponding RGB image.

To test the applicability of our findings we conducted another experiment aiming at a more practical use. Cucumber (*Cucumis sativa*) plants were manually infected with mildew (*Spaerotheca fugliginea*) as visible by an infested area at the leaf apex and randomly distributed spots on the rest of the leaf (Figure 10). Infested leaves were scanned with our scanning device using exposure times ranging from 5 to 2,100. Whereas at exposure times of 80 and higher, most of the surface of the leaf was imaged, at lower exposure times surface structures such as leaf veins and the mildew infested areas became visible (Figure 10). Particularly with an exposure time of 10 infested leaf areas could be easily identified.

## 4. Discussion

Our scanning system, a laser scanner coupled to an articulated measuring arm (Figure 2), is a quite advantageous configuration for volumetric scans, because it supports an automated fusion of different points of view together with a high resolution and a high accuracy. To standardise our results we used a consistent angle of incidence of approximately 90°. However, small deviations due to manual movement of the sensors cannot be ruled out.

We tried to conserve the conditions of our experiments regarding the illumination to attribute occurring inaccuracies and effects to the laser scanner itself. It is very likely that using the measuring combination under different conditions like e.g., ambient light will be without further effects due to the sensor's integrated chromatic filter and the amount of sunlight that is, compared to the laser energy, very small. Current state-of-the-art outdoor 3D sensors use a similar laser energy to measure distances of more than hundred meters with highest accuracy. However, this has to be proven directly by further studies before any ultimate statement can be made.

In our measurement a value in the form of a 3D point was either recorded or the measurement was automatically discarded, resulting in a missing pixel. The rejection of data is the consequence of a fixed internal sensor setting for the minimum required intensity on the sensor array and the maximum allowed width of the laser line on the CCD chip. Ignoring these limits would result in measurements that cannot be separated from the sensor's intrinsic noisiness or errors resulting from incoming ambient light, respectively. Especially in the field of close-up laser scanning, where the intended resolution and the actual possible accuracy are in the same order of magnitude, these influencing factors would compromise the acquisition of correct data. However, the sensor does not support to gain insight about the actual reason for rejection of data and hence it is unknown if a pixel was rejected due to a low intensity of the received light or because the signal was too blurred, *i.e.*, the line of received light was too wide. In any case, the rejection of such data contributes to the accuracy of the measurements.

Nevertheless, despite of the internal quality check of the sensor clearly false pixels were recorded, when measuring multiple layers of epidermal strips (Figure 9) or the transition from green to white areas (Figure 3). In both cases this was only observed at high exposure times. This indicates systematic errors which are probably caused by reflection and scattering of the incoming laser light by the underlying glass slide (Figure 8) or the leaf tissue (Figure 3). Inaccurate 3D measurements observed at the transition from light to dark can be explained by the fact directly on the border the laser beam simultaneously covers a light and a dark area. This is comparable to the so-called edge effect [8,11].

We observed additional effects that, under some conditions, prevented the complete and/or correct imaging of plant structures. These effects were caused by absorption of the light emitted with a wavelength of 660 nm by chlorophyll. This wavelength is common in commercially available state-of- the-art laser scanning devices [13,14] and especially in the field of combined laser scanning devices [6] as used in industrial applications. Whereas the standard wavelength of 660 nm is well suited for commercial applications, such as quality testing in automotive industry, but it may be problematic in plant imaging if the laser light reaches the chlorophyll-containing mesophyll [15]. If this is the case, the incoming light may be absorbed resulting in missing pixels at low exposure times, as it was observed in our study in *F. benjamina* (Figures 3 and 4), *P. laurocerasus* (Figure 6) and with filter paper impregnated with chlorophyll (Figure 5). In these cases it was possible to image the objects by increasing the exposure time. These findings are consistent with [8], who found an impact of the chlorophyll content on the intensity of the reflected laser signal for terrestrial laser scanners using a time of flight measuring technique. If these effects influence the signals for low resolution long range scans they require particular attention for high resolution close-up laser scanning.

Whereas in the aforementioned experiments low exposure times turned out to be disadvantageous, the opposite was true in the experiments with *A. porrum*. Here, with a low exposure time, the most accurate imaging was obtained and we found evidence that the recorded images represented the epidermis as the outermost cell layer if not the leaf surface itself (Figures 8 and 9). The reason for the different behaviour of *A. porrum* leaf surfaces is unknown, but it is very likely that the high surface roughness resulting from the structured epidermis and the dense coverage with epicuticular waxes led to an increased reflection of the incoming laser light and thus to a lower proportion of light that was transmitted by the epidermis. This conclusion is additionally supported by our observations with mildew-infested leaves of *C. sativa*. Here, at low exposure times the rough surfaces of the mildew infested patches were imaged whereas the rather smooth areas surrounding the patches were not (Figure 10), most likely due to light absorption by chlorophyll. These findings are comparable to those of [16]. They found a significant change of the spectral reflectance of healthy sugar beet leaves and those infected with powdery mildew, especially in the area of the visible light.

We found indications of a strong effect of the chlorophyll concentration of leaves on the images obtained with different exposure time. On the one hand, this is direct evidence that in this case the recorded pixels do not originate from the leaf surface but at least partly from the leaf interior. This is a major disadvantage, if the aim is to correctly image the true plant geometry as it is important for parameter extraction such as leaf or stem volume. On the other hand, we obtained different images of the leaf interior as a function of exposure time and conducted some preliminary experiments to test this as a tool for the detection of plant health. These experiments gave first indications that it may be possible to classify leaves or leaf areas according to their visibility at different exposure times and thereby to differentiate between mildew infected and non-infected areas (Figure 10) or between different stages of leaf senescence (Figures 6 and 7). This is in accordance with [17] and [18], who found a clear separable reflection behaviour for chlorotic leaves or diseases changing the surface structure (mildew).

Similarly, [14] distinguished leaves with different physiological states by their laser reflection properties. However, they used four different laser wavelengths and a resolution that was much lower than in our experiments.

## 5. Conclusions

Aiming at reliable and accurate laser scans of plant surfaces, we showed the various effects caused by laser penetration and absorption in different plant tissues. We used a standard measuring device for close-up laser scanning at 660 nm and demonstrated the influence of epidermis thickness, surface roughness, chlorophyll concentration of the mesophyll, as well as of the phenology and plant diseases on the scanning result.

We found that the signal received by the sensor is, independent of the exposure time, a mixed signal coming from different depths of the leaf tissue, depending on its optical properties. The exposure time enables controlling the depth range the 3D coordinate is derived from by widening the laser line with higher exposure times. Uncertain signals, as recorded at certain exposure times, were discarded due to the sensors implemented thresholds.

Our results define a research gap on the influence of plant tissue properties on measurements of standard laser scanning devices. This interaction has to be further studied, aiming at a highly accurate and reliable 3D model of a plant derived by laser scans and measuring smallest deformations of plant organs due to environmental changes like stress. To minimize interferences caused by leaf chlorophyll, a green laser would be superior to the common red laser. However, even with a green laser reducing the absorption of the laser beam, the problems caused by the transparency of the epidermis, border effects or refraction of light causing blurred sensor images would probably still persist.

Additional research should focus on developing situationally adapted camera exposure times which are currently only available for the scanning of technical objects and a validation of the influence of external light sources for application in outdoor use. This would enable highly precise 3D models of living plants, which would however still be less accurate than with non-biological surfaces. Despite these constraints, laser triangulation provides the highest accuracy and the highest resolution of all currently available 3D measuring techniques for the close-up imaging of plants.
